# Paeonol could alleviate diabetes‐related spermatogenic dysfunction via SIRT3‐dependent redox rebalancing

**DOI:** 10.1002/ctm2.1585

**Published:** 2024-02-15

**Authors:** Man Liu, Xintao Gao, Mengying Shi, Min Li, Taotao Sun, Jiaxin Wang, Wenchao Xu, Bai Jian, Jihong Liu, Xiaming Liu, Delin Ma, Yi Wang

**Affiliations:** ^1^ Department of Urology Tongji Hospital Tongji Medical College Huazhong University of Science and Technology Wuhan China; ^2^ Department of Geriatrics Tongji Hospital Tongji Medical College Huazhong University of Science and Technology Wuhan China; ^3^ Department of Urology Sir RunRun Shaw Hospital College of Medicine Zhejiang University Hangzhou China; ^4^ College of Pharmaceutical Sciences Zhejiang University Hangzhou China; ^5^ Reproductive Medicine Center Tongji Hospital Tongji Medical College Huazhong University of Science and Technology Wuhan Hubei China; ^6^ Department of Endocrinology Tongji Hospital Tongji Medical College Huazhong University of Science and Technology Wuhan Hubei China; ^7^ National Key Laboratory of Chinese Medicine Modernization, Innovation Center of Yangtze River Delta, Zhejiang University Jiaxing China

Dear Editor,

Diabetes seriously impairs male reproductive function, and its adverse effects on male fertility need to be addressed, with treatment options being explored.^1^ The overproduction of reactive oxygen species (ROS) is the main mechanism of diabetes affecting male reproductive health.^2^, ^3^, ^4^ Our study provides evidence for the critical role of sirtuin 3 (SIRT3) in mediating the effect of paeonol on spermatogenesis and a potential therapeutic option for spermatogenesis disorders in diabetic patients.

The type 1 diabetes rat model was used to observe testicular atrophy and subsequently examined changes in sperm quality. Our results showed that the level of SIRT3 was significantly reduced and the level of acetylation in testicular tissues was elevated (Figure S1).

To assess the protective role of SIRT3 in sperm quality, we employed systemic *Sirt3* knockout mice (Figure S2A). The *Sirt3* knockout mice exhibited unaltered testis and seminiferous tubule morphology but decreased sperm quality (Figures 1A,B and S2B–E). Furthermore, the results revealed that *Sirt3* knockout prompted elevated oxidative stress and apoptosis of germ cells across all stages in the seminiferous tubules, along with amplified DNA damage, possibly connected to the redox homeostasis that SIRT3 helps maintain (Figures 1C–E and S2F–J). SIRT3 regulated ROS levels by activating antioxidant enzymes through direct deacetylation of superoxide dismutase 2 (SOD2) and interacting with FOXO3A (Figure 1F).^5^ We found similar results in vitro by using high glucose (Figure S3). High glucose injury resulted in decreased SIRT3 levels and boosted Ac‐SOD2 expression, and the increase in ROS and apoptosis levels was alleviated by the upregulation of SIRT3 levels (Figure 1G–I). These results suggest that SIRT3‐mediated redox balance is indispensable for maintaining the process of spermatogenesis.

**FIGURE 1:**
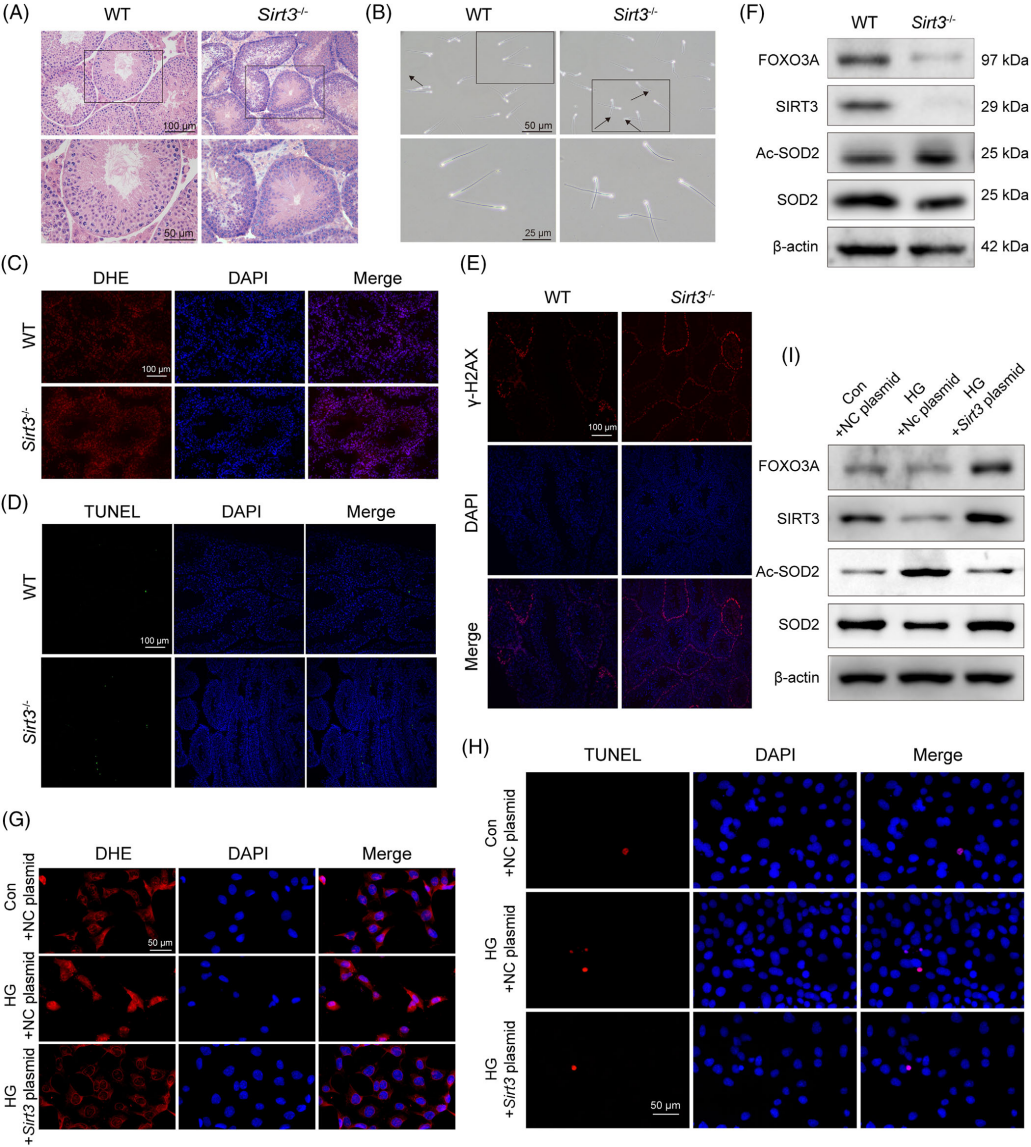
Role of SIRT3‐mediated redox homeostasis in spermatogenesis. (A) Representative testicular H&E sections. (B) Morphological observation of spermatozoa under the microscope (black arrows indicate deformed spermatozoa). (C) Representative pictures and quantification of dihydroethidium (DHE) staining (ROS, red; 4′,6‐diamidino‐2‐phenylindole (DAPI), blue), *n* = 3. (D) Representative pictures and quantification of TUNEL staining (apoptosis‐positive, green; DAPI, blue), *n* = 3. (E) Representative pictures and quantification of γ‐H2AX fluorescent staining in testis tissue (γ‐H2AX, red; DAPI, blue), *n* = 3. (F) The expression of FOXO3A, SIRT3, SOD2 and Ac‐SOD2 proteins in testis tissue was evaluated by Western blotting, *n* = 3. (G) Representative pictures and quantification of DHE staining (ROS, red; DAPI, blue), *n* = 3. (H) Representative pictures and quantification of TUNEL staining (TUNEL positive cell, red; DAPI, blue), *n* = 5. (I) After overexpressing SIRT3, the expression of FOXO3A, SIRT3, SOD2, and Ac‐SOD2 proteins in cells was evaluated by Western blotting, *n* = 3. The scale bars are marked on the figure, average ± SD, *P<.05, **P<.01, ****P<.0001.

We focused on natural plant components to explore new effective drugs^6^ and found several monomeric compounds from peony bark, which have significant effects on antidiabetic and antioxidant stress.^7^ We examined the effects of these compounds on the GC‐1 cell model treated with high glucose through a drug screen (Figure S4A), which showed that only paeonol significantly increased SIRT3 expression, especially at the concentration of 10 μM (Figures 2A–D and S4B–C). In addition, cell proliferation and viability were inhibited at higher concentrations of paeonol (Figure S4D). In our study, we performed molecular docking analysis to investigate the interaction between paeonol and NAD+. Our results demonstrated that paeonol has the ability to bind to the hydrophobic residues of SIRT3 by utilizing its π‐system. Additionally, paeonol formed hydrogen bonds (H‐bonds) with Ile 230 and ASP231, further enhancing the binding affinity (Figure S4E). These results suggest that paeonol can increase SIRT3 levels in high‐glucose treated GC‐1 cells.

**FIGURE 2:**
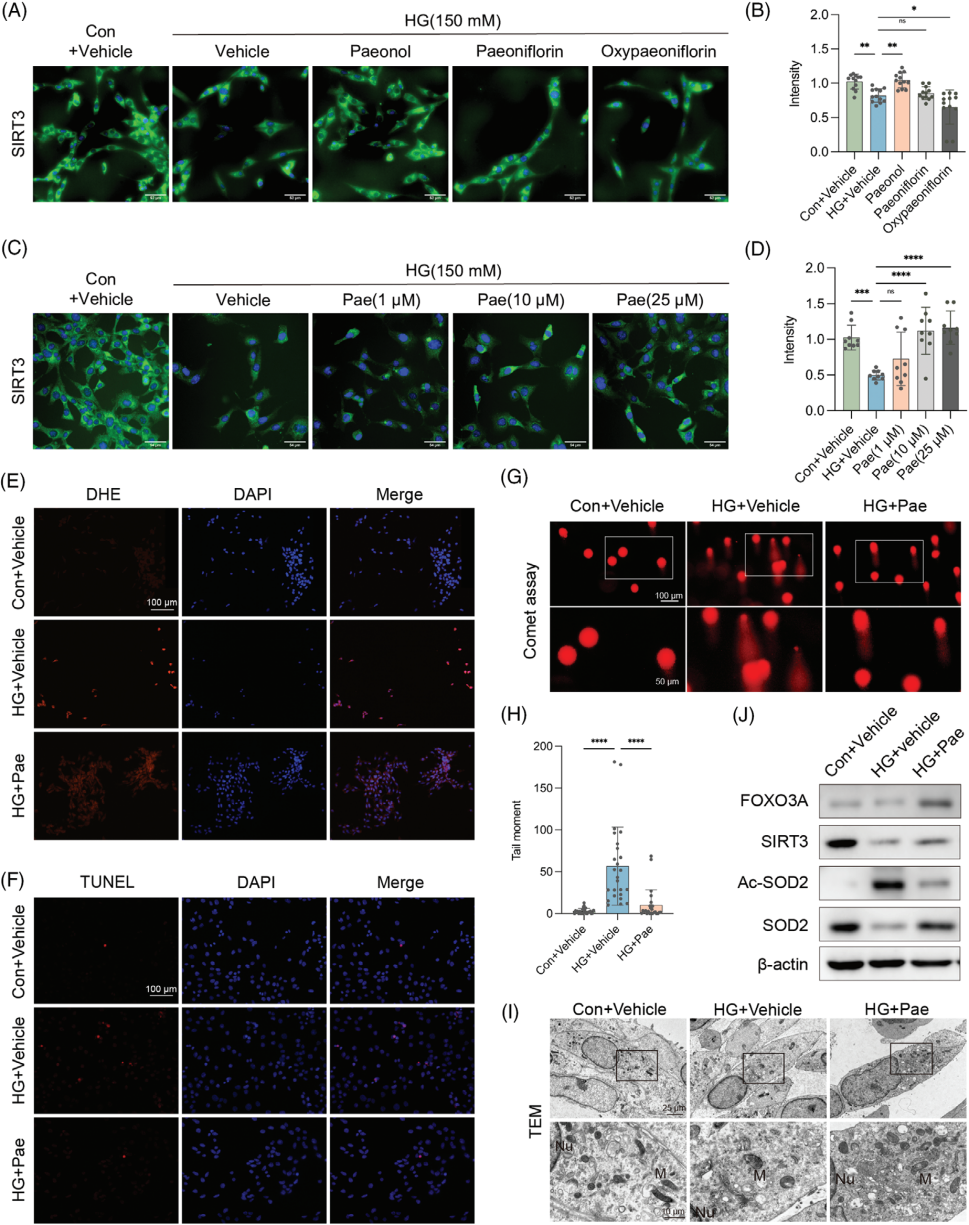
Screening of possible effective monomer compounds from peony bark and verification of antioxidant effect in vitro. (A,B) Confocal representative images and quantification of the effects of paeonol, paeoniflorin, and oxypaeoniflorin on SIRT3 expression. (C,D) Confocal representative pictures and quantification of the effect of different concentrations of paeonol on the expression of SIRT3. (E) Representative pictures and quantification of DHE staining (ROS, red; DAPI, blue), *n* = 3. (F) TUNEL staining to evaluate the level of apoptosis, representative pictures, and quantification (TUNEL positive cell, red; DAPI, blue), *n* = 5. (G,H) Comet assay to assess DNA damage, representative pictures (DNA, red). (I) The mitochondrial changes of GC‐1 cells observed by transmission electron microscopy after paeonol intervention, representative pictures (N, nucleus; M, mitochondria). (J) After paeonol intervention, the expression of FOXO3A, SIRT3, SOD2, and Ac‐SOD2 proteins in cells was evaluated by Western blot, *n* = 3. The scale bars are marked on the figure, average ± SD, *P<.05, **P<.01, ***P<.001, ****P<.0001.

We proceeded to validate the protective impact of paeonol (Figure S5). Paeonol administration resulted in reduced ROS levels and fewer TUNEL‐positive cells following high glucose exposure (Figures 2E,F and S5B–E). The paeonol treatment group exhibited a significant decrease in the proportion and tail moment of comet tail DNA (Figure 2G,H). SIRT3 is mainly located in mitochondria, which is also an important site for redox reactions. We observed that paeonol intervention ameliorated the impairment of mitochondrial number and morphology in the high‐glycemic group (Figure 2I). Moreover, paeonol rescued high glucose‐induced SIRT3 downregulation and increased SOD2 activity (Figures 2J and S5I). Together, these findings suggest that paeonol can alleviate excessive oxidative stress caused by high glucose in vitro and is linked to SIRT3‐involved antioxidant responses.

Similar testicular damage was observed in diabetic rat models (Figures 3A–F and S6). Feeding paeonol decreased the ROS level in testis tissue of diabetic rats (Figure 3H). TUNEL results revealed that paeonol lowered the apoptosis level of spermatogenic cells at all seminiferous tubule levels, which was consistent with the Bax and Bcl‐2 ratio (Figure 3J). Furthermore, paeonol improved DNA damage in response to γ‐H2AX (Figure 3I). The transmission electron microscopy results demonstrated that the mitochondria in spermatogonia were condensed, disintegrated, and reduced in number in the diabetic group. After paeonol intervention, the mitochondria morphology of spermatogonia recovered, and a clear lamellar shape with observable crests was observed (Figure 3K). Finally, we examined the effect of paeonol on the SIRT3 antioxidant pathway in vivo, and the results demonstrated that paeonol rescued diabetes‐induced SIRT3 downregulation and increased SOD2 activity (Figure 3G). In summary, paeonol protected the testis from diabetes‐induced damage through SIRT3‐mediated antioxidant effects, improving sperm quality in diabetic rats.

**FIGURE 3:**
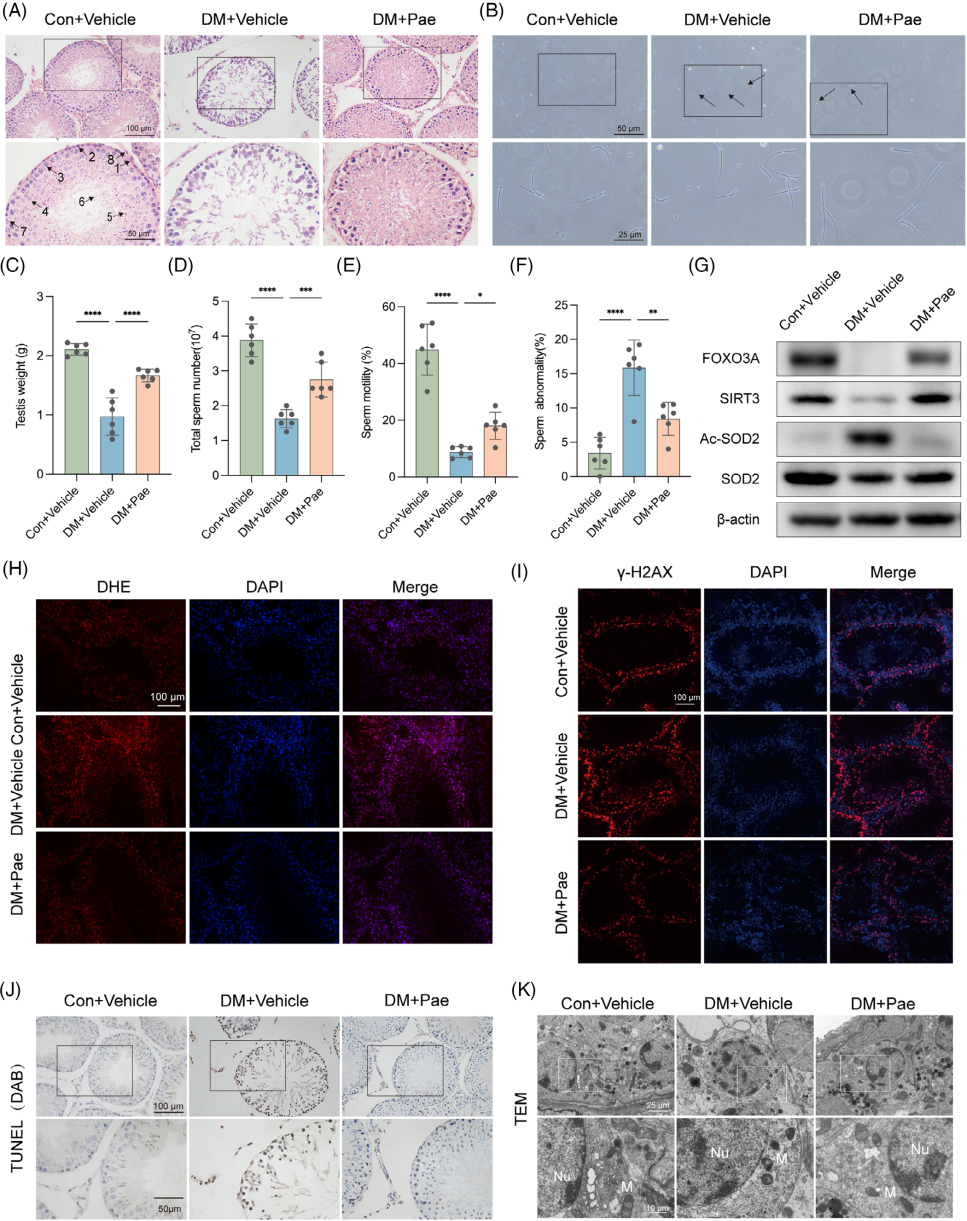
Effect of paeonol on sperm quality in diabetic rats. (A) Representative testicular H&E sections (1: myoid cells; 2: spermatogonia; 3: primary spermatocytes; 4: secondary spermatocytes; 5: spermatids; 6: spermatozoa; 7: supporting cells; 8: Leydig cells;). (B) Morphological observation of spermatozoa under the microscope (the black arrow points to abnormal spermatozoa). (C) Weight of unilateral testis, *n* = 6. (D) The total number of spermatozoa in one testis, *n* = 6. (E) Sperm motility, *n* = 6. (F) Sperm deformity rate, *n* = 10. (G) After paeonol intervention, the expression of FOXO3A, SIRT3, SOD2, and Ac‐SOD2 proteins in testis tissue was evaluated by Western blot, *n* = 3. (H) Representative pictures and quantification of DHE staining (ROS, red; DAPI, blue) of testis tissue, *n* = 3. (I) Representative pictures and quantification of γ‐H2AX fluorescent staining in testis tissue (γ‐H2AX, red; DAPI, blue), *n* = 3. (J) TUNEL staining to evaluate the level of apoptosis in testis tissue, representative pictures, and quantification (apoptosis‐positive cell, brown). (K) Transmission electron microscope observation of mitochondrial changes in spermatogonia after paeonol intervention, representative pictures (N, nucleus; M, mitochondria). The scale bars are marked on the figure, average ± SD, *P<.05, **P<.01, ***P<.001, ****P<.0001.

Diabetes may affect the process of spermatogenesis by increasing oxidative stress, destroying supporting cells, and producing excessive glycation end products, etc., and we focused on oxidative stress. SIRT3 is mainly expressed in mitochondria which plays a crucial role in protecting tissues against oxidative stress damage. In asthenoteratozoospermic men, lower levels of SIRT3 in seminal plasma were negatively correlated with oxidative stress and DNA fragmentation in semen.^8^ Previous studies have explored the antioxidant effects of paeonol, and it can improve the pathological damage of diabetic encephalopathy, increase rat body weight, and reduce blood glucose levels.^9^ This inconsistency may be due to the fact that the type 1 diabetic rats in this study had higher blood sugar levels and more severe damage, which provides evidence that paeonol improved sperm quality through the anti‐oxidative stress effect rather than blood sugar regulation. Then, our screening of monomeric compounds in peony bark was based on the activation of SIRT3. Since diabetes can easily lead to erectile dysfunction, which can affect mating behavior, all animal experiments focused on sperm quality and did not test the fertility of male animals.^10^

In conclusion, our findings suggest that SIRT3 is a crucial mediator of impaired spermatogenesis in diabetes. Paeonol, a natural compound, exerts its reproductive protective effect by enhancing the antioxidant capacity mediated by SIRT3/SOD2 signalling pathway, potentially providing a new therapeutic avenue for diabetic infertility patients.

## AUTHOR CONTRIBUTIONS

Xiaming Liu, Delin Ma, and Yi Wang designed this study. Man Liu, Xintao Gao, and Mengying Shi performed the study. Man Liu and Xintao Gao wrote the manuscript. Taotao Sun, Jiaxin Wang, Min Li, Bai Jian, Jihong Liu, and Wenchao Xu contributed to the discussion. Man Liu and Xintao Gao analysed the results.

## CONFLICT OF INTEREST STATEMENT

The authors declare no conflicts of interest.

## ETHICAL APPROVAL

The study involving animal subjects was approved by the Institutional Animal Care and Use Committee (IACUC) of Tongji Hospital, Protocol ID: TJH‐202010010.

## Supporting information


**Figure S1** Diminished sperm quality observed in diabetic rats. (A) Graphical summary of the results in this section. (B) Timeline of diabetic rat modelling. Blood glucose (C) and body weight (D) of rats in the control group and DM group before and after enrollment, *n* = 4. Testis weight (E), total sperm count (F), sperm motility (G), and sperm deformity rate (H) of rats in the control group and the DM group, *n* = 4. (I) Representative testicular H&E sections (1: myoid cells; 2: spermatogonia; 3: primary spermatocytes; 4: secondary spermatocytes; 5: spermatids; 6: spermatozoa; 7: Sertoli cell; 8: Leydig cells). (J) Morphological observation of spermatozoa under a microscope (black arrows indicate deformed spermatozoa). (K,L) The expression of FOXO3A, SIRT3, and Acely‐Lysine proteins in testis tissue was evaluated by the Western blot. The scale bars are marked on the figure, average ± SD, *P<.05, **P<.01, ***P<.001, ****P<.0001.

**Figure S2** SIRT3‐mediated redox homeostasis is crucial for spermatogenesis. (A) qRT‐PCR detection of Sirt3 mRNA level in testis tissue, *n* = 3. (B) Weight of unilateral testis, *n* = 10. (C) The total number of sperm in one testis, *n* = 10. (D) Sperm motility, *n* = 10. (E). Sperm deformity rate, *n* = 10. (F) Quantification of DHE staining (ROS, red; DAPI, blue), *n* = 3. (G) Quantification of TUNEL staining (apoptosis‐positive, green; DAPI, blue), *n* = 3. (H) Quantification of γ‐H2AX fluorescent staining in testis tissue (γ‐H2AX, red; DAPI, blue), *n* = 3. (I,J) The expression of apoptosis‐related proteins Bax and Bcl‐2 in testis tissue was assessed by Western blotting, *n* = 3. (K) The relative expression of SOD2 and Ac‐SOD2 proteins in testis tissue was evaluated, *n* = 3. The scale bars are marked on the figure, average ± SD, *P<.05, **P<.01, ****P<.0001.

**Figure S3** Role of SIRT3‐mediated redox homeostasis in vitro experiment. (A) Grouping and intervention scheme of in vitro experiment. (B) The ratio of Ac‐SOD2 and SOD2 proteins in cells was evaluated by Western blotting, *n* = 3. (C) Quantification of DHE staining (ROS, red; DAPI, blue), *n* = 3. (D) Quantification of TUNEL staining (TUNEL positive cell, red; DAPI, blue), *n* = 5. (E,F) After overexpression of *Sirt3*, the expression of Bax and Bcl‐2 proteins was assessed by Western blot, *n* = 3. The scale bars are marked on the figure, average ± SD, *P<.05, ***P<.001.

**Figure S4** The process of screening out paeonol. (A) The method and process of drug screening. (B,C) SIRT3 protein levels were detected by Western blot (concentrations of paeonol:.1, 1, 10, 25 μM). (D) The CCK8 method was used to detect the effect of paeonol intervention at different concentrations and at different times on cell viability. (E) The molecular docking results. The scale bars are marked on the figure, average ± SD, **P<.01, ****P<.0001.

**Figure S5** Supplementary quantitative results of vitro experiments. (A) Grouping and intervention scheme of in vitro experiment. (B) Quantification of DHE staining (ROS, red; DAPI, blue), *n* = 3. (C,D) The effect of paeonol on the antioxidant capacity and lipid peroxidation level, using kits to detect SOD activity and MDA content, *n* = 3. (E) Quantification of TUNEL staining to evaluate the level of apoptosis (TUNEL positive cell, red; DAPI, blue), *n* = 5. (F,G) After paeonol intervention, the expression of Bax and Bcl‐2 proteins was assessed by Western blot, *n* = 3. (H) Comet assay to assess DNA damage, representative pictures (DNA, red). (H) Represent the DNA content in tail and tail moment, *n* = 25. (I) After paeonol intervention, the relative expression of SOD2 and Ac‐SOD2 proteins in cells. The scale bars are marked on the figure, average ± SD, **P<.01, ***P<.001, ****P<.0001.

**Figure S6** Supplementary results of the effect of paeonol in diabetic rats. (A) Timeline of diabetic rat modelling and paeonol administration intervention. Blood glucose (B) and body weight (C) of rats in each group before, during, and after the experiment, *n* = 6. (D) Quantification of DHE staining (ROS, red; DAPI, blue) of testis tissue, *n* = 3. (E) and (F) After paeonol intervention, the expression of Bax and Bcl‐2 proteins in testis tissues was evaluated by Western blot, *n* = 3. (G) Quantification of γ‐H2AX fluorescent staining in testis tissue (γ‐H2AX, red; DAPI, blue), *n* = 3. (H) After paeonol intervention, the relative expression of SOD2 and Ac‐SOD2 proteins in testis tissue. The scale bars are marked on the figure, average ± SD, *P<.05, ****P<.0001.

## Data Availability

The authors confirm that the data supporting the findings of this study are available within the letter and its Supporting information.
