# Supplementary figures and images for: Paeonol could alleviate diabetes‐related spermatogenic dysfunction via SIRT3‐dependent redox rebalancing
Source: Clin Transl Med. 2024 Feb 15;14(2):e1585. doi: 10.1002/ctm2.1585 (PMC10867591; doi:10.1002/ctm2.1585)

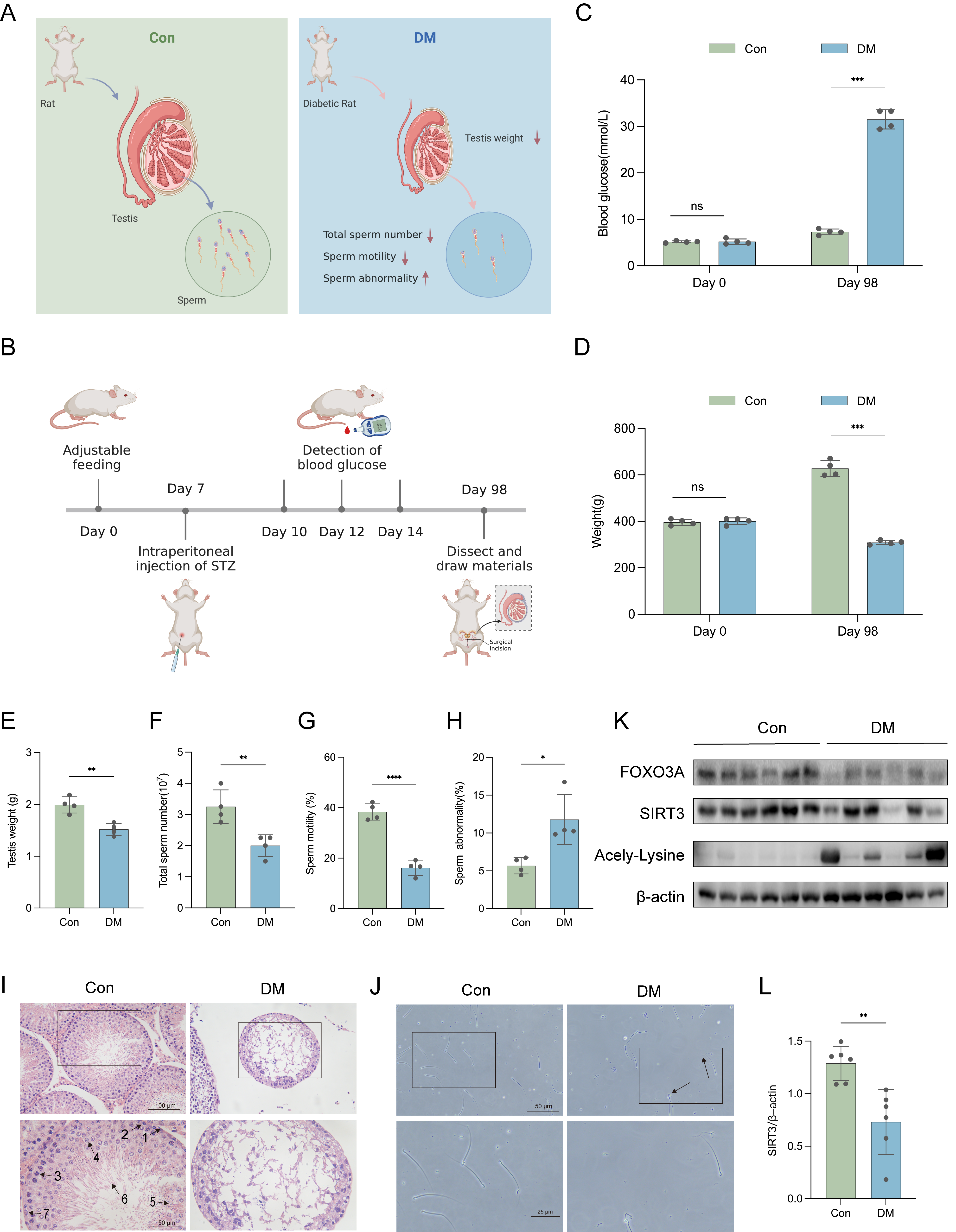

Supplement: Supplementary file 1 — Figure S1 Diminished sperm quality observed in diabetic rats. (A) Graphical summary of the results in this section. (B) Timeline of diabetic rat modelling. Blood glucose (C) and body weight (D) of rats in the control group and DM group before and after enrollment, n = 4. Testis weight (E), total sperm count (F), sperm motility (G), and sperm deformity rate (H) of rats in the control group and the DM group, n = 4. (I) Representative testicular H&E sections (1: myoid cells; 2: spermatogonia; 3: primary spermatocytes; 4: secondary spermatocytes; 5: spermatids; 6: spermatozoa; 7: Sertoli cell; 8: Leydig cells). (J) Morphological observation of spermatozoa under a microscope (black arrows indicate deformed spermatozoa). (K,L) The expression of FOXO3A, SIRT3, and Acely‐Lysine proteins in testis tissue was evaluated by the Western blot. The scale bars are marked on the figure, average ± SD, *P<.05, **P<.01, ***P<.001, ****P<.0001. [file CTM2-14-e1585-s006.tif]

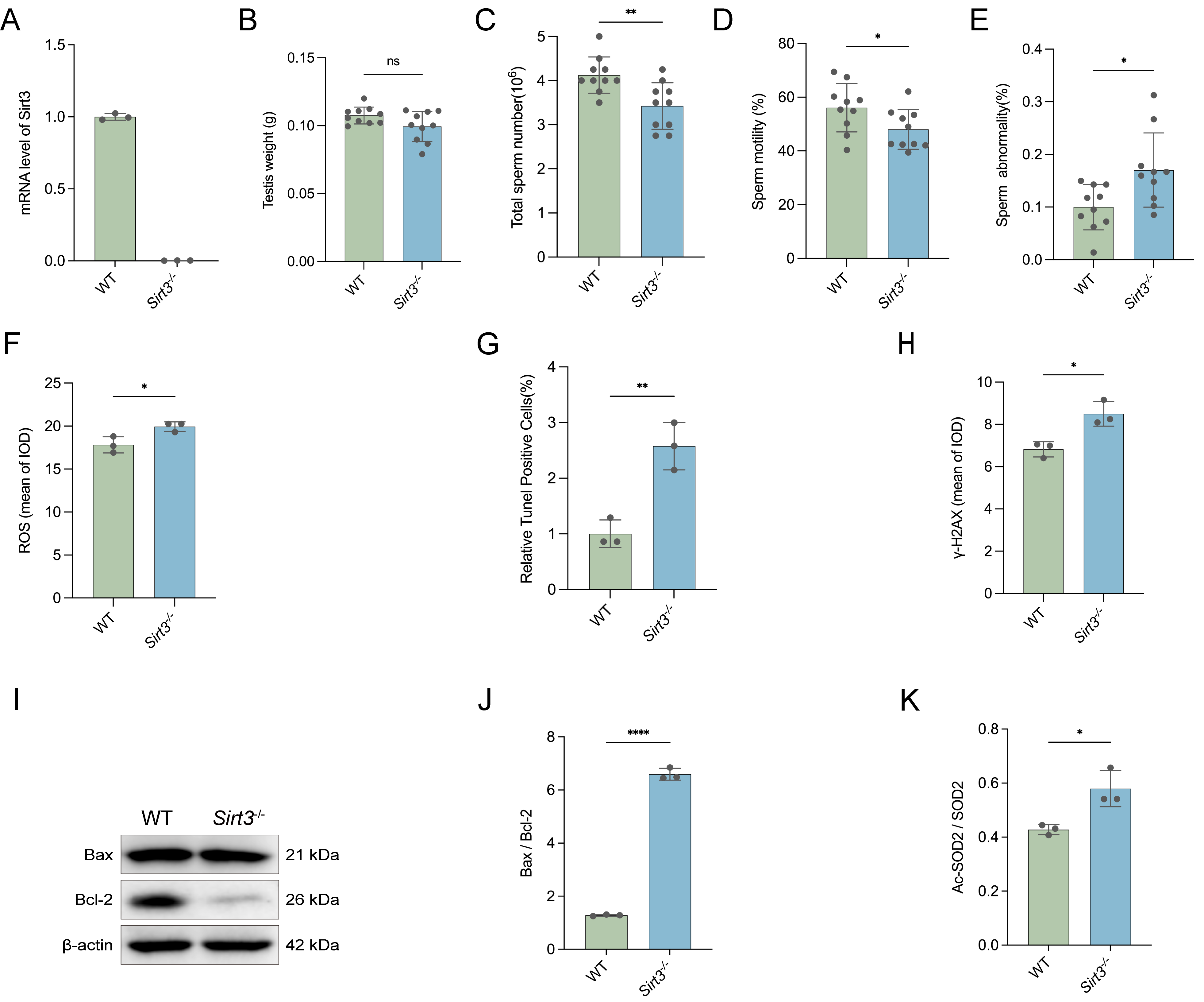

Supplement: Supplementary file 2 — Figure S2 SIRT3‐mediated redox homeostasis is crucial for spermatogenesis. (A) qRT‐PCR detection of Sirt3 mRNA level in testis tissue, n = 3. (B) Weight of unilateral testis, n = 10. (C) The total number of sperm in one testis, n = 10. (D) Sperm motility, n = 10. (E). Sperm deformity rate, n = 10. (F) Quantification of DHE staining (ROS, red; DAPI, blue), n = 3. (G) Quantification of TUNEL staining (apoptosis‐positive, green; DAPI, blue), n = 3. (H) Quantification of γ‐H2AX fluorescent staining in testis tissue (γ‐H2AX, red; DAPI, blue), n = 3. (I,J) The expression of apoptosis‐related proteins Bax and Bcl‐2 in testis tissue was assessed by Western blotting, n = 3. (K) The relative expression of SOD2 and Ac‐SOD2 proteins in testis tissue was evaluated, n = 3. The scale bars are marked on the figure, average ± SD, *P<.05, **P<.01, ****P<.0001. [file CTM2-14-e1585-s001.tif]

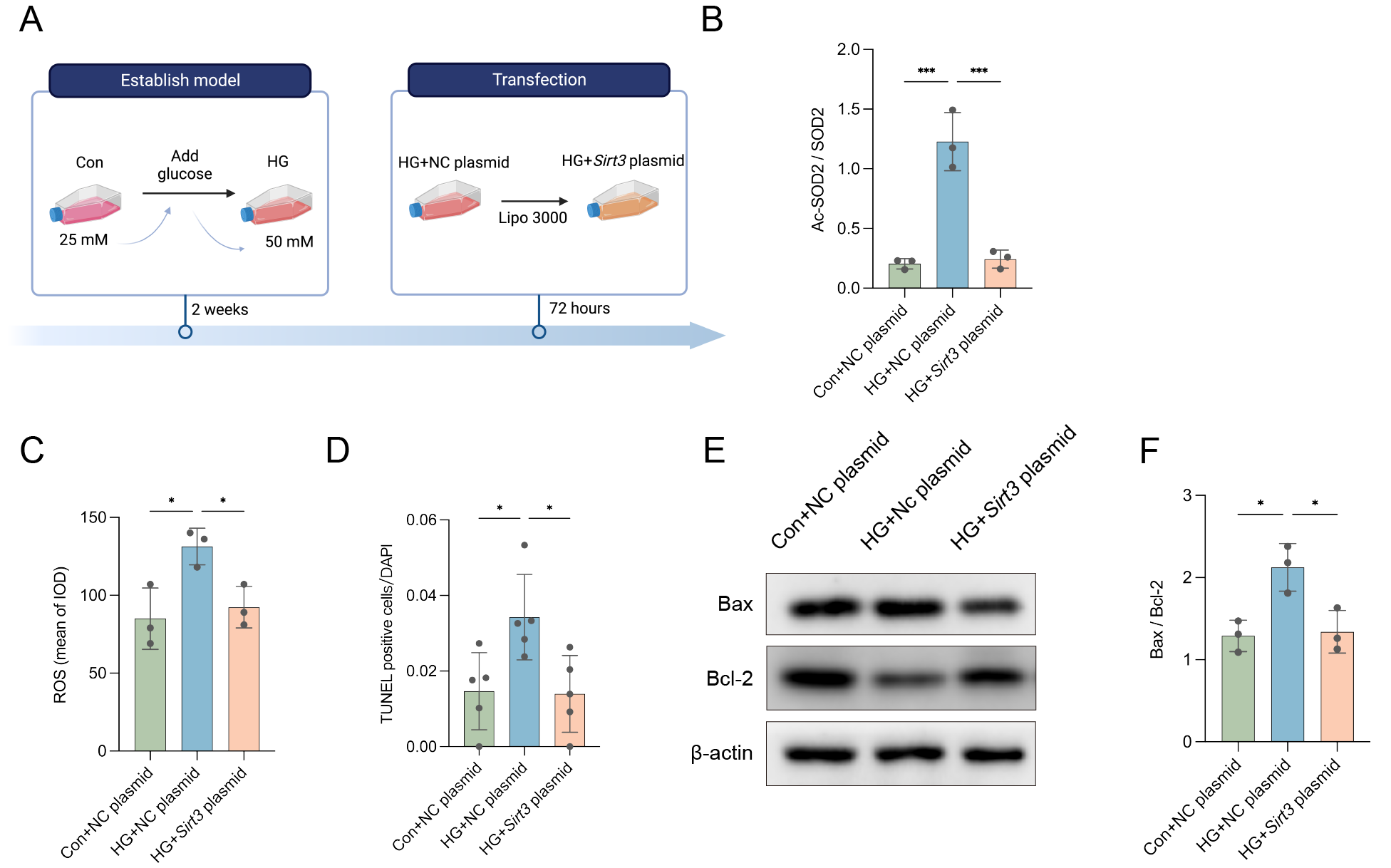

Supplement: Supplementary file 3 — Figure S3 Role of SIRT3‐mediated redox homeostasis in vitro experiment. (A) Grouping and intervention scheme of in vitro experiment. (B) The ratio of Ac‐SOD2 and SOD2 proteins in cells was evaluated by Western blotting, n = 3. (C) Quantification of DHE staining (ROS, red; DAPI, blue), n = 3. (D) Quantification of TUNEL staining (TUNEL positive cell, red; DAPI, blue), n = 5. (E,F) After overexpression of Sirt3, the expression of Bax and Bcl‐2 proteins was assessed by Western blot, n = 3. The scale bars are marked on the figure, average ± SD, *P<.05, ***P<.001. [file CTM2-14-e1585-s003.tif]

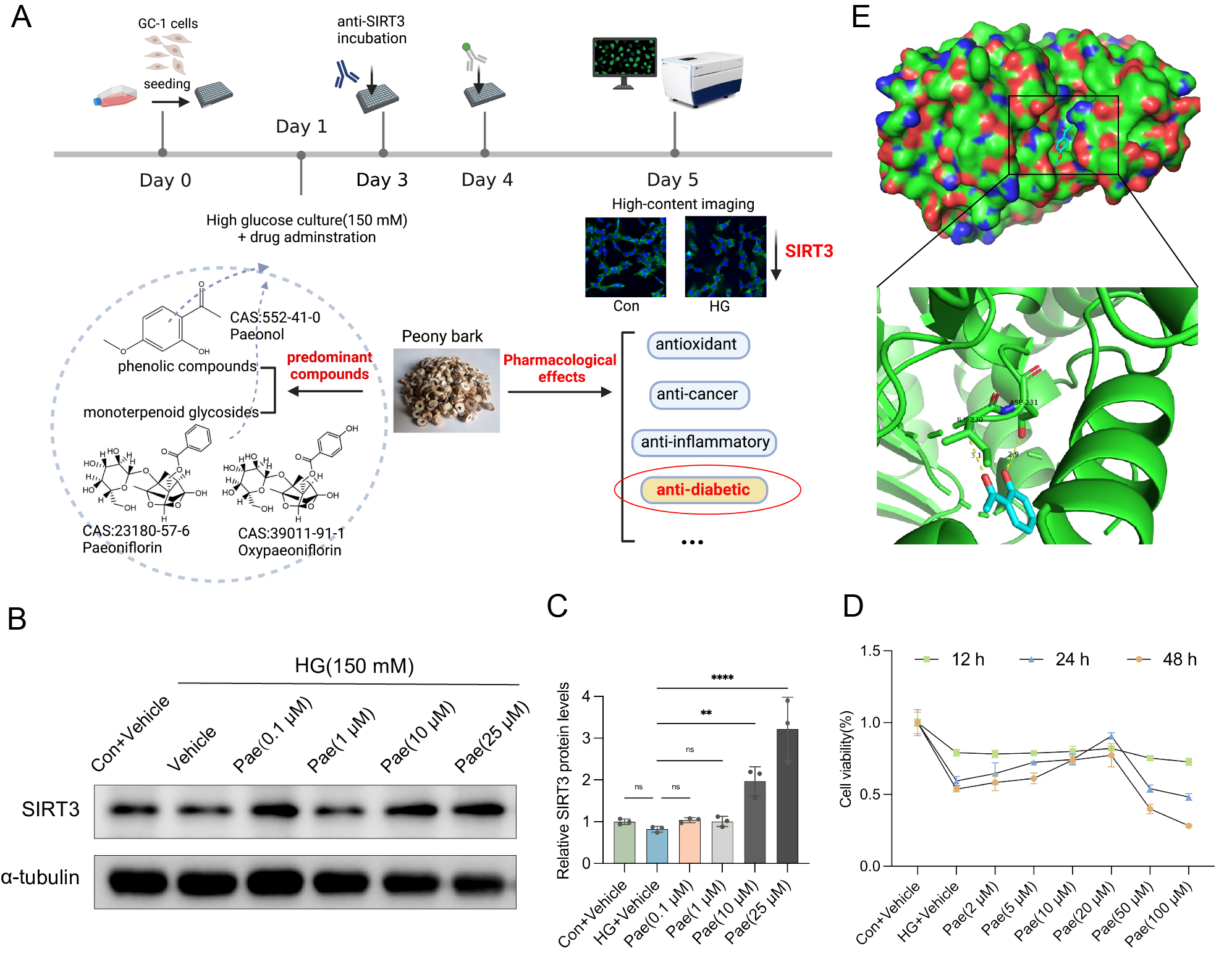

Supplement: Supplementary file 4 — Figure S4 The process of screening out paeonol. (A) The method and process of drug screening. (B,C) SIRT3 protein levels were detected by Western blot (concentrations of paeonol:.1, 1, 10, 25 μM). (D) The CCK8 method was used to detect the effect of paeonol intervention at different concentrations and at different times on cell viability. (E) The molecular docking results. The scale bars are marked on the figure, average ± SD, **P<.01, ****P<.0001. [file CTM2-14-e1585-s002.tif]

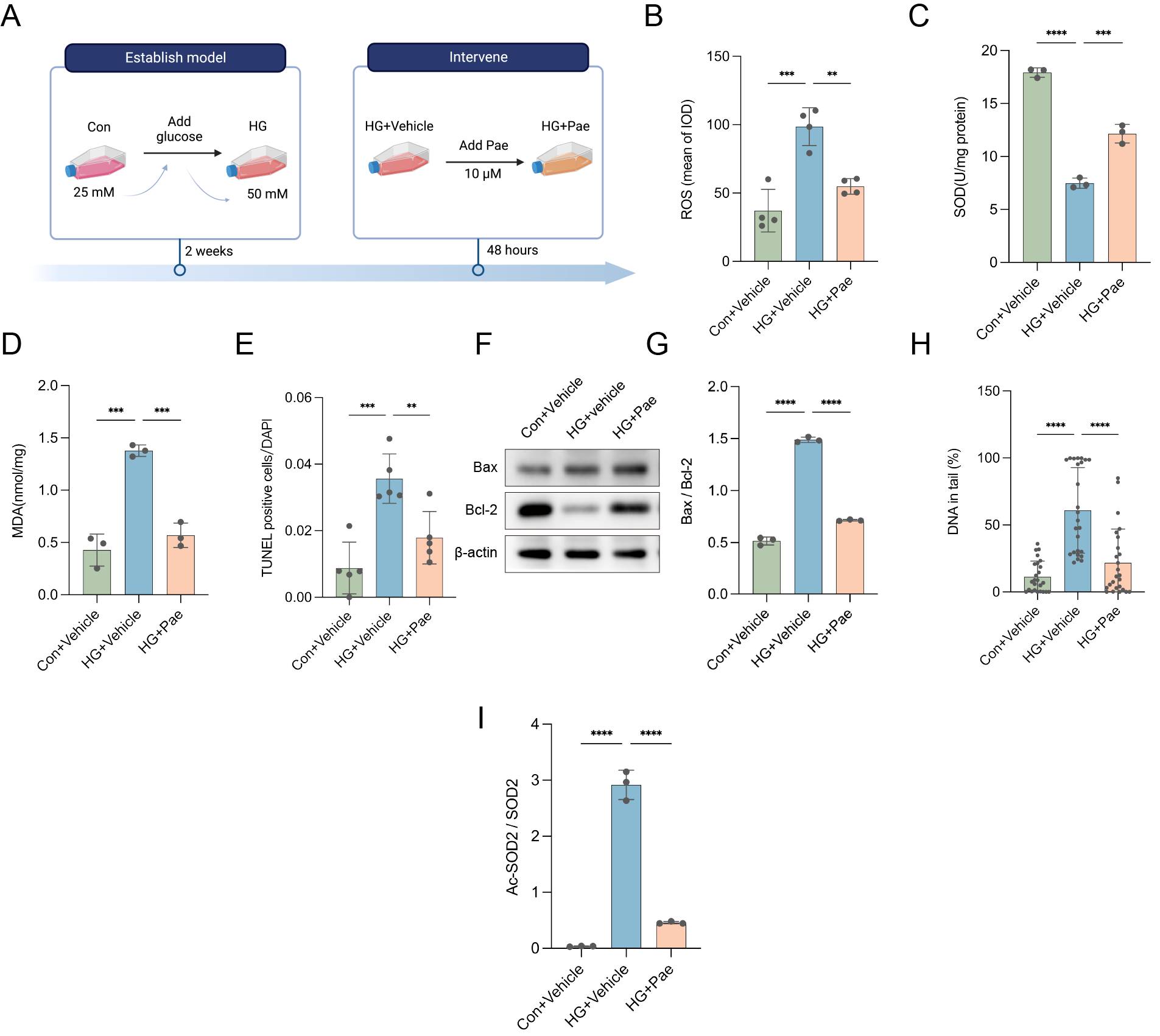

Supplement: Supplementary file 5 — Figure S5 Supplementary quantitative results of vitro experiments. (A) Grouping and intervention scheme of in vitro experiment. (B) Quantification of DHE staining (ROS, red; DAPI, blue), n = 3. (C,D) The effect of paeonol on the antioxidant capacity and lipid peroxidation level, using kits to detect SOD activity and MDA content, n = 3. (E) Quantification of TUNEL staining to evaluate the level of apoptosis (TUNEL positive cell, red; DAPI, blue), n = 5. (F,G) After paeonol intervention, the expression of Bax and Bcl‐2 proteins was assessed by Western blot, n = 3. (H) Comet assay to assess DNA damage, representative pictures (DNA, red). (H) Represent the DNA content in tail and tail moment, n = 25. (I) After paeonol intervention, the relative expression of SOD2 and Ac‐SOD2 proteins in cells. The scale bars are marked on the figure, average ± SD, **P<.01, ***P<.001, ****P<.0001. [file CTM2-14-e1585-s004.tif]

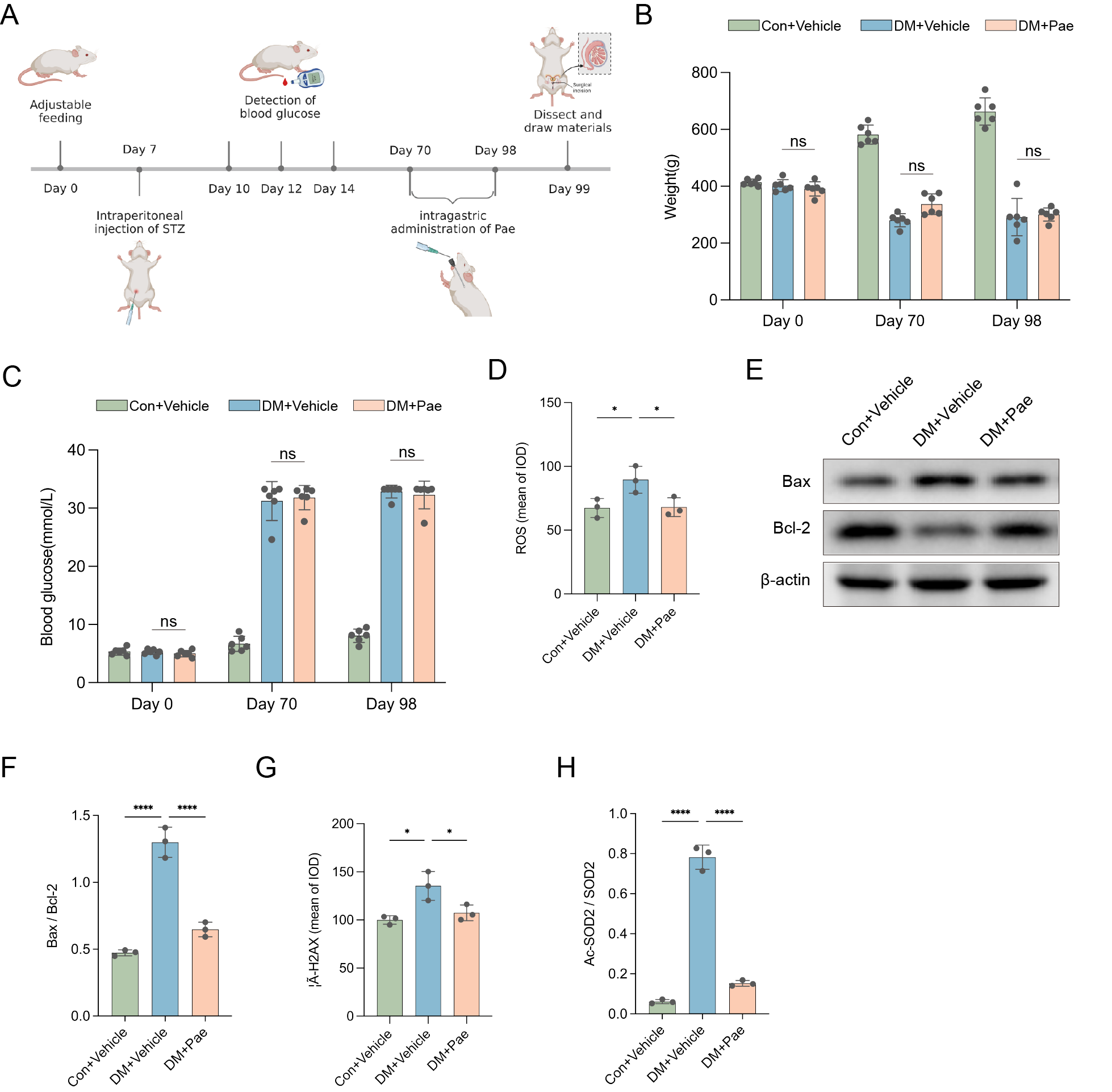

Supplement: Supplementary file 6 — Figure S6 Supplementary results of the effect of paeonol in diabetic rats. (A) Timeline of diabetic rat modelling and paeonol administration intervention. Blood glucose (B) and body weight (C) of rats in each group before, during, and after the experiment, n = 6. (D) Quantification of DHE staining (ROS, red; DAPI, blue) of testis tissue, n = 3. (E) and (F) After paeonol intervention, the expression of Bax and Bcl‐2 proteins in testis tissues was evaluated by Western blot, n = 3. (G) Quantification of γ‐H2AX fluorescent staining in testis tissue (γ‐H2AX, red; DAPI, blue), n = 3. (H) After paeonol intervention, the relative expression of SOD2 and Ac‐SOD2 proteins in testis tissue. The scale bars are marked on the figure, average ± SD, *P<.05, ****P<.0001. [file CTM2-14-e1585-s005.tif]
